# Delay discounting decisions are linked to temporal distance representations of world events across cultures

**DOI:** 10.1038/s41598-020-69700-w

**Published:** 2020-07-31

**Authors:** Denise E. Croote, Baojun Lai, Jingchu Hu, Mark G. Baxter, Alison Montagrin, Daniela Schiller

**Affiliations:** 1 The Nash Family Department of Neuroscience, Icahn School of Medicine at Mount Sinai, New York, NY 10029 USA; 2 Department of Psychiatry, Icahn School of Medicine at Mount Sinai, New York, NY 10029 USA; 3 School of Psychology and Center for Studies of Psychological Application, South China Normal University, Guangzhou, 510631 China

**Keywords:** Decision, Human behaviour

## Abstract

Delay discounting describes the phenomenon whereby the subjective value of a reward declines as the time until its receipt increases. Individuals differ in the subjective value that they assign to future rewards, yet, the components feeding into this appraisal of value remain unclear. We examined whether temporal psychological distance, i.e. the closeness one feels to the past and future, is one such component. English speakers in the USA and Mandarin speakers in China completed a delay discounting task and organized past and future world events on a canvas according to their representation of the event’s temporal position relative to themselves. Previous work has identified linguistic and cultural differences in time conception between these populations, thus, we hypothesized that this sample would display the variability necessary to probe whether temporal psychological distance plays a role in reward valuation. We found that English speakers employed horizontal, linear representations of world events, while Mandarin speakers used more two-dimensional, circular representations. Across cultures, individuals who represented the future as more distant discounted future rewards more strongly. Distance representations of past events, however, were associated with discounting behaviors selectively in Mandarin speakers. This suggests that temporal psychological distance plays a fundamental role in farsighted decision-making.

## Introduction

Time is highly abstract and intangible in that it cannot be seen, touched, heard or sensed directly, yet it is an integral component of daily decision-making. When choosing between attending a concert tonight or a weekend getaway next month, one must weigh the value of each option against the backdrop of time. Does one month feel as though it is right around the corner, or a decade away? In general, humans prefer immediate rewards to delayed rewards, a phenomenon known as temporal discounting^1,2^. Previous research has shown, however, that individuals vary substantially in the degree to which they discount future rewards. Variability in discounting behavior has been associated with socioeconomic status^3^, feelings of self-continuity^4,5^, and several psychiatric disorders^6–10^. Neural activity in the ventral striatum, medial prefrontal cortex, and posterior cingulate cortex has already been shown to track subjective value during temporal discounting^1^, but the key factors that feed into this construction of value are not well understood.

Individuals’ subjective perception of the time delay to receive a reward is one factor that has been shown to play a role in temporal decision-making^11–14^. This line of work suggests individuals do not accurately represent objective changes in duration and instead represent future time horizons nonlinearly^11^. For instance, though 12 months is objectively twice as long as 6 months, it is often perceived to be less than two times as long^12^. In the current study, we focus on the role of past and future psychological distance in guiding discounting decisions, rather than the nonlinear perception of prospective temporal delays. Psychological distance details more generally the extent to which something diverges from ones direct existence^15^. Psychological distance is egocentric in nature and contains the additional elements of spatial, social, and hypothetical distance. Here we concentrate selectively on the temporal dimension, which describes the experienced closeness of the past and future^16,17^. We first quantify temporal distance representations for real world events across individuals and we then investigate whether distance representations explain differences in discounting behaviors.

To parse out individual differences, we sought to obtain a sample with a range of temporal representations. We recruited English and Mandarin speakers due to substantial evidence suggesting that North Americans and East Asians differ in how they conceptualize time. Speakers of these languages differ in their use of space–time metaphors, where English speakers’ reference time using mostly horizontal spatial metaphors, like *back* when I was a kid, or in the weeks *ahead*^18,19^. Mandarin speakers also utilize horizontal spatial metaphors. However, vertical metaphors are used more systematically in Mandarin than in English, where *shàng* (up, top) refers to events earlier in time and *xià* (down, bottom) events later in time^18,20^. In English, the past is linguistically mapped as behind the self and the future as ahead of the self. In Mandarin by contrast, *qián* (front) is used to reference the past/before, while *hòu* (back) is used reference the future/after^21^. Further, the presence of tenses in English promotes temporal segmentation by clearly distinguishing what has, what is, and what will happen, while the absence of explicit tenses in Mandarin fosters temporal continuity^22^. Previous work has also shown that North Americans predict linear trends and focus on shorter, more stable time horizons, while East Asians predict cyclical patterns and consider longer time horizons^23^. Lastly, East Asians have a stronger past orientation than North Americans, where the past has been shown to play a larger role in explaining current events, motivating behaviors, and constructing ones identity in East Asian culture^23^. Collectively, we hypothesized that this cross-cultural sample would display a range of temporal representations, which in turn would allow us to delineate whether individual differences in temporal psychological distance correlate with differences in discounting behavior.

**Figure 1:**
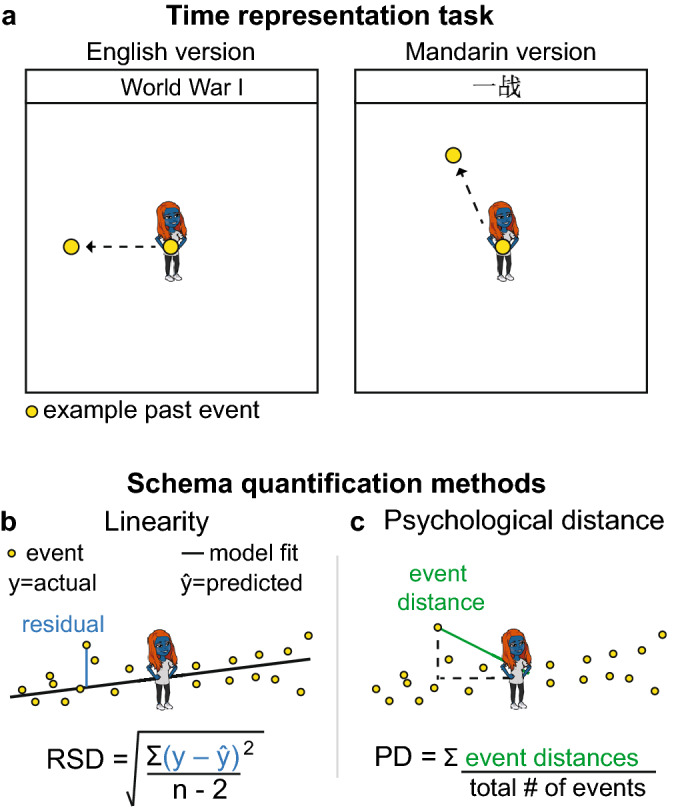
Time representation task. (**a**) Participants placed 72 past and future events on an XY canvas to reflect *when* they felt the event occurred or could occur in relation to themselves. (**b**) Linear model fit to all event placements. Residuals represented the difference between actual (y) and predicted (ŷ) placements, and the residual standard deviation (RSD) summarized the degree to which a linear model appropriately characterized the event schema. (**c**) Psychological distance (PD) calculated as the average of all of the vectors connecting each event to the center avatar.

**Figure 2:**
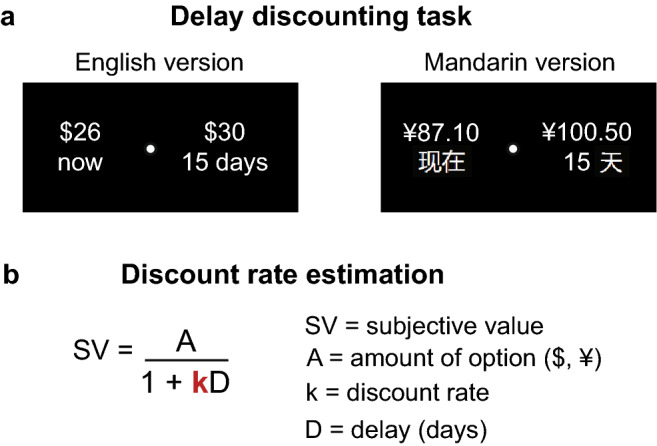
Delay discounting task. (**a**) Participants chose between receiving less money now or more money after a variable delay. (**b**) Participants’ responses were modeled using the hyperbolic function, SV = A/(1 + kD), where a higher discount rate k indicated that a participant more steeply discounted future rewards.

We developed a novel time representation task to examine how English and Mandarin speakers schematically represent past and future world events in relation to themselves (Fig. 1a). Participants were shown a blank canvas and 72 internationally known past and future world events in their native language. They were signified by a blue avatar in the center and asked to drag-and-drop the events anywhere on the canvas to reflect *when* they felt the event occurred or could occur in relation to themselves. We assessed the general schemas employed to represent the temporal events using a measure of linearity (Fig. 1b) and quantified the psychological distance with which the events were represented from the self (Fig. 1c). We paired this task with a delay discounting task (Fig. 2a), where participants indicated their preferences for a lesser amount of money now or a larger amount of money after a delay, and we quantified participants’ willingness to wait for future rewards using the discount rate k (Fig. 2b).

We hypothesized that individuals who represented temporal events as further from themselves would be less willing to wait for future rewards. Events included both past and future occurrences, and we were specifically interested in examining the role of distance representations of each timeframe in the valuation of future rewards. Episodic memory and episodic foresight, or future simulation, engage the same core network of brain regions, including the hippocampus, parahippocampal cortex, and the temporal and inferior posterior parietal cortices^24^. Past memories shape our personal narrative^25^ and enable us to predict, simulate, and plan for future outcomes^17,26^. Further, manipulating participants’ representation of the future by enhancing its perceived concreteness with detail^4,27,28^ and cueing individuals to engage in episodic foresight can alter discounting behavior^29–31^. Given these anatomical and functional connections, we hypothesized that distance representations of both the past and future would influence discounting behavior. However, temporal focus on the past and the role of the past in directing behaviors has been shown to differ between East Asians and North Americans^23^. Therefore, we sought examine whether the relationship between psychological distance and discounting behavior differed as a function of timeframe and culture.

## Results

### Mandarin speakers more readily use the vertical dimension when representing world events

Forty native English speakers in the United States (M = 23.5 years, SD = 2.75, n = 22 female) and 40 Mandarin speakers in China (M = 23.0 years, SD = 3.05, n = 20 female) completed the experimental tasks. There was no difference in average age across the cultures (Wilcoxon sum rank test; W = 919.5, two-tailed, *P* = 0.25, 95% CI for the difference in location [− 1.00, 2.00]) and the order in which the tasks were completed was counterbalanced in each sample. Time representation events were selected from a larger pool of 184 world events based on survey results from an independent sample of 30 English and 30 Mandarin speakers (see methods). Results were filtered to remove events that were considered too unrealistic or unfamiliar and the final 72 events were balanced on familiarity, arousal, and valence across cultures. We included historic and futuristic events from within and beyond participants’ lifespans to examine how participants abstractly represented themselves in a grander temporal context^32^. Participants were shown an event and asked to place it anywhere on the canvas to reflect *when* they felt it occurred or could occur in relation to themselves and the [x,y] grid-coordinates of each event placement were recorded.

**Figure 3:**
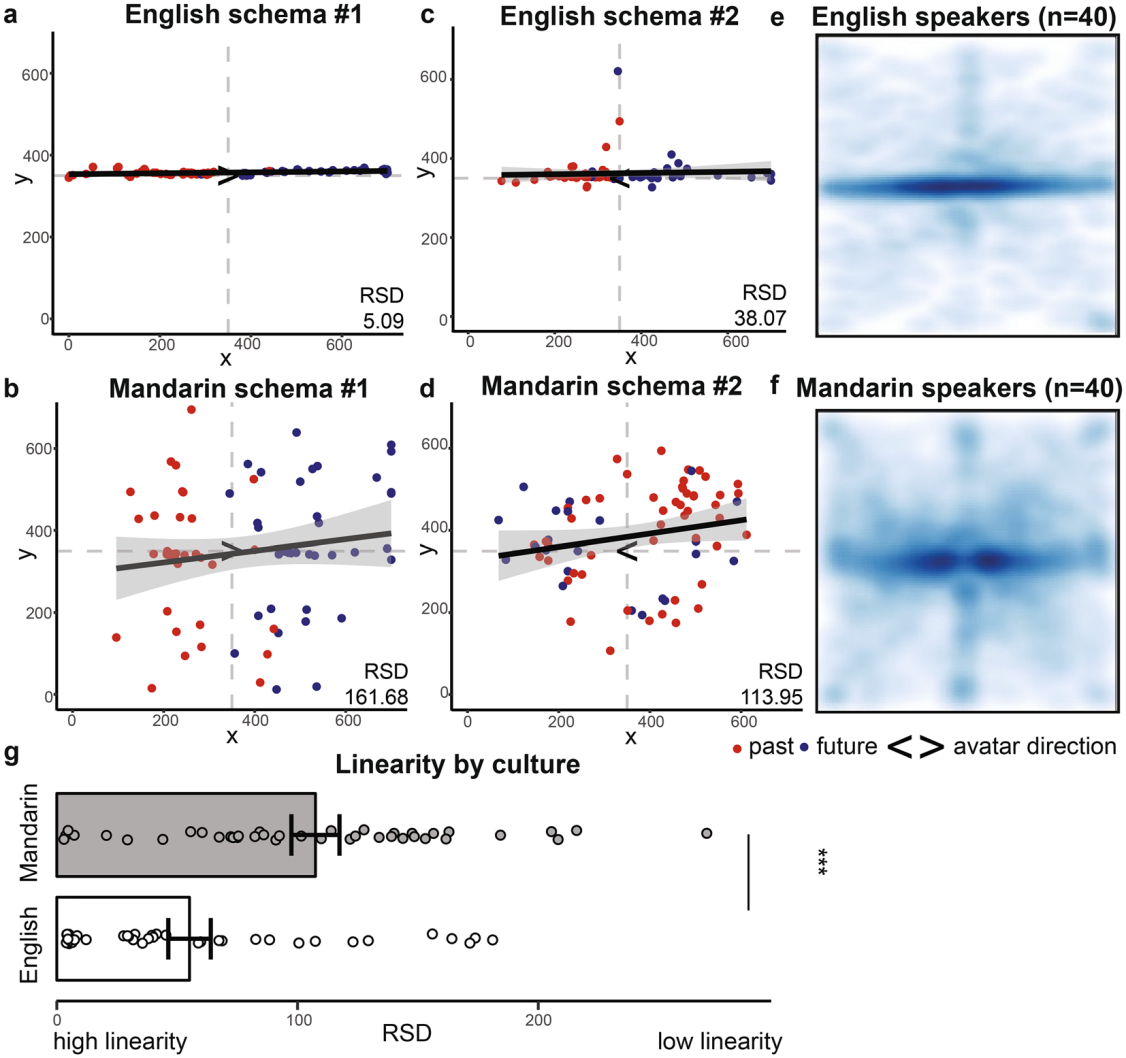
Mandarin speakers more readily use the vertical dimension when representing world events. Linear model fit and resultant residual standard deviation (RSD) for example English and Mandarin speakers’ time representation schemas. (**a**,**b**) depict participants with more extreme and (**c**,**d**) more median RSD scores in their respective cultures. Density plots for event placements across all (**e**) English and (**f**) Mandarin speakers. (**g**) English speakers employed significantly more linear schemas (lower RSD) than Mandarin speakers (Wilcoxon sum rank test: W = 430, ***P < 0.001). Error bars = SEM.

We found that English speakers conceptualized time using largely horizontal, one-dimensional timelines, while Mandarin speakers plotted events using both the horizontal and vertical dimensions, leading to qualitatively more circular representations. To quantify participants’ space–time schemas, we applied a measure of linearity. We fit a linear model to each participant’s 72 event placements and extracted the residual standard deviation (RSD) of the model (Fig. 1b). The residuals captured the differences between the observed and predicted event placements and the RSD summarized the degree to which a linear model appropriately characterized the event schema. Though there was variability in each sample, linear regressions largely captured English speakers’ schemas well, producing small RSDs (M = 55.12, SEM = 8.82, Mdn = 36.82; see example participants in Fig. 3a,c), while models of Mandarin speakers’ schemas produced larger RSDs (M = 107.44, SEM = 9.99, Mdn = 105.70; see example participants in Fig. 3b,d). Density plots for all participants further depict the linear schemas used by English speakers (Fig. 3e), when compared to the more dimensionally distributed schemas employed by Mandarin speakers (Fig. 3f). When comparing across cultures, English speakers placed events significantly more linearly (Fig. 3g, Wilcoxon rank sum test; W = 430, two-tailed, *P* < 0.001, 95% CI for the difference in location [− 81.52, − 28.31], Cohen’s d = 0.88).

Following the time representation task, participants rated each event on its arousal, familiarity, and valence using a sliding scale of 1–7. When accounting for mean event familiarity, arousal, and valence, and participant age, sex, and education using multiple linear regression, the relationship between linearity and culture persisted (F_(1,71)_ = 10.54, *P* = 0.002, β = 57.23, *η*^2^ = 0.12, model R^2^ = 0.20). Statistical significance was evaluated using Type III Sums of Squares. We then explored whether the vertical placement of events observed in Mandarin speakers followed a temporal gradient, with events earlier in time being placed above events later in time, as would be predicted by the vertical metaphors present in Mandarin. We calculated a rank correlation between the true temporal distance of past events and their placement along the Y axis for each participant. We observed a weak, but significant positive correlation across Mandarin speakers, which indicates that participants tended to place more distant past events above more recent past events (Supplementary Fig. S1). Speakers varied, however, in the degree to which they adhered to this temporal gradient and further study is needed to identify the additional parameters influencing placements along the vertical dimension. In summary, we found that when controlling for demographic factors and event ratings, Mandarin speakers employed less linear schemas and more readily used the vertical dimension when representing world events. Within the space–time schemas employed by each participant, we were specifically interested in examining temporal psychological distance.

### Strong individual variability in temporal distance representations and discount rates

**Figure 4:**
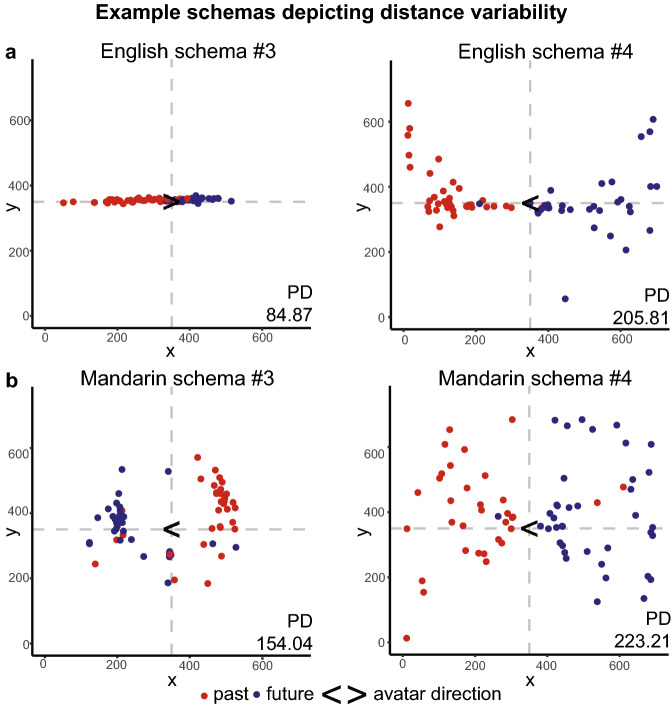
Example schemas depict the large variability in temporal distance representations present across participants. Event schemas for two (**a**) English and two (**b**) Mandarin speakers. Events colored as past (red) or future (blue) according to the participants’ ratings of its timeframe.

**Figure 5:**
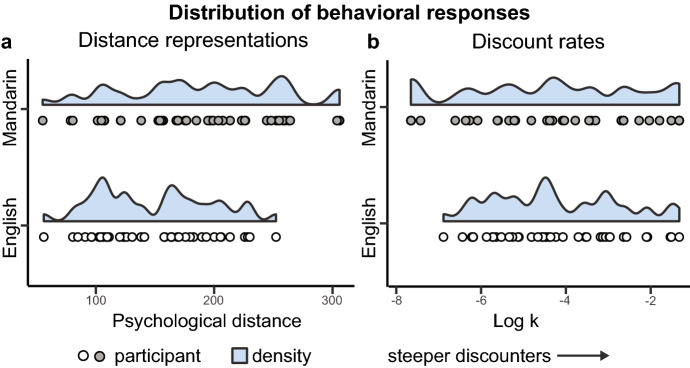
Strong individual variability in distance representations and discount rates across cultures. (**a**) Distribution of distance representations for English (n = 40) and Mandarin (n = 40) speakers. (**b**) Distribution of log transformed discount rates for English (n = 39) and Mandarin (n = 35) speakers.

We observed strong individual variability in both temporal distance representations and discount rates within our samples. On the time representation task, the distance between each event placement and the avatar’s center was represented by the length of the vector connecting the two points. For each participant, we captured his or her represented closeness of time by averaging the 72 event distances into an individual psychological distance (PD) value (Fig. 1c). While some participants concentrated events in close proximity of their avatar, others extended placements to the edges of the canvas. This was apparent regardless of whether a participant employed a more horizontal or dimensionally distributed schema (see individual examples in Fig. 4a,b). Altogether, participants in both cultures substantially varied in the degree to which they utilized the canvas space (Fig. 5a).

On the delay discounting task, participant’s responses were modeled using the hyperbolic function, SV = A/(1 + kD), where a higher discount rate k indicated that a participant more steeply discounted future rewards (Fig. 2b)^33,34^. We assessed the goodness-of-fit of the models using McFadden’s pseudo-R^2^ and excluded participants whose R^2^ fell below 0.3, a threshold set to exclude participants for responding randomly^7,10,35^. The final sample consisted of 74 participants, 39 of which were English and 35 of which were Mandarin speaking (McFadden’s R^2^: Mdn = 0.72, range = 0.33–1.0). One Mandarin speaker chose all immediate rewards and three Mandarin speakers chose all delayed rewards. Discount rates for these participants were constrained to the highest and lowest values that could be reliably estimated in the Mandarin-speaking sample^7^. Discount rates were not normally distributed and were natural log transformed prior to completing all statistical analyses^10^. As expected, we observed significant individual variability in discounting behaviors within each culture (Fig. 5b).

Following the time representation task, participants rated the date range of each event (further past, closer past, closer future, or further future). Due to the abstract nature of the task and our focus on subjective PD, events were categorized and visualized as past or future based on the individual participants’ evaluation of its timeframe in all analyses. We did not observe any differences in the percentages of events rated in each of the four timeframes, suggesting that the cultures did not differ in their semantic categorizations of the events (Supplementary Fig. S2). Moreover, to validate that our task was capturing subjective PD, we examined whether events rated as occurring further in time were indeed placed farther from the avatar than events rated as occurring closer in time. In both English and Mandarin speakers, further past and further future-rated events were placed significantly farther from the avatar than closer past and closer future-rated events respectively (Supplementary Fig. S2).

We also dissected our PD value into the average distances for events that each participant rated as occurring in the future and in the past. Across cultures, Mandarin speakers had significantly larger average PD scores than English speakers for both future and past events (Supplementary Fig. S3, Future: Mandarin: M = 196.96, SEM = 10.39, English: M = 148.84, SEM = 8.61, Two Sample t-test; t_(78)_ = 3.56, two-tailed, *P* < 0.001, 95% CI [21.25, 75.00], Past: Mandarin: M = 179.88, SEM = 11.23, English: M = 150.83, SEM = 8.26, Two Sample t-test; t_(78)_ = 2.08, two-tailed, *P* = 0.040, 95% CI [1.31, 56.80]). We did not observe a difference in the average PD of past and future events within English speakers, alternatively, Mandarin speakers asymmetrically represented past-rated events as significantly closer to themselves than future-rated events (Supplementary Fig. S3). Further, we did not observe a difference in log transformed discount rate, log k, by culture (Two Sample t-test; t_(72)_ = 0.39, two-tailed, *P* = 0.70, 95% CI for the difference in means [− 0.66, 0.98]). Nor did we observe a difference in log k based on the order in which the experiment was completed, i.e. whether participants completed the time representation task first and discounting task second or the converse (Supplementary Fig. S4).

Most importantly, there were no differences in the variance of past and future distance representations across cultures, the variance of past and future distance representations within each culture, or the variance in discount rates across cultures (Supplementary Table S1). Past and future distance representations were correlated within each culture, however, the strength of the correlations did not statistically differ across cultures (Supplementary Table S1). In summary, we observed a spectrum of behavioral responses on each paradigm and the variability present in both cultures optimally positioned us to examine the relationship between PD representations and discount rates.

### Distance representations of world events correlate with discount rates

To examine the relationship between distance representations for world events and log transformed discount rates we used a series of linear mixed models. More specifically, we examined whether the relationship between individual event distances and discount rate varied as a function of culture (English speaking vs. Mandarin speaking) or timeframe (past vs. future). Event distances were coded as the dependent variable and the participants’ discount rates as the predictor. This design allowed us to retain all event distance evaluations per participant, rather than average these distances into a single value. We included culturally adjusted income bracket, socioeconomic status, financial security, age, sex, and education as fixed factors in our models and events and participants as random factors. Events and participants were coded as random factors to account for the lack of independence between the placement of an event across participants (e.g., different participants may place “World War I” similarly), and the lack of independence between the 72 event placements within a participant (see Supplementary Table S2 for full formulas). We used the lme4^36^ and lmerTest^37^ packages in R v. 3.6.0^38^ and evaluated statistical significance using Type III Sums of Squares.

**Figure 6:**
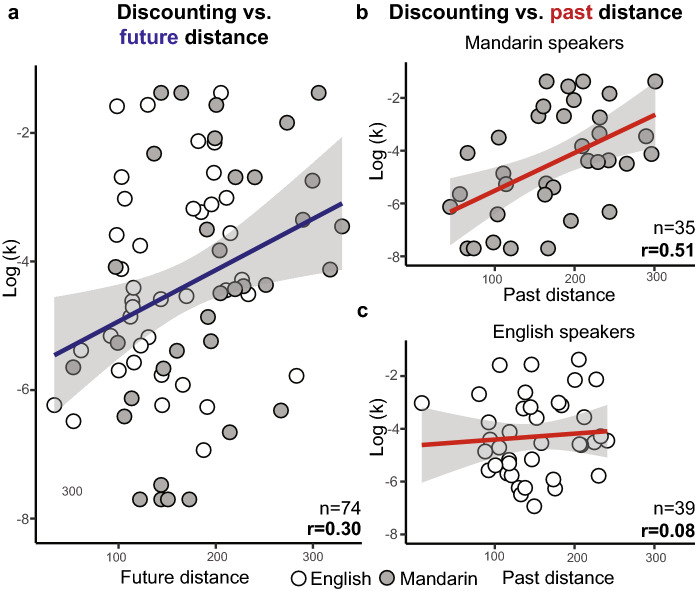
Distance representations of world events correlate with discount rates. (**a**) Significant positive correlation between average PD for future events and log k across cultures, Pearson’s r = 0.30, t_(72)_ = 2.67, *P* = 0.009, n = 74. (**b**) Significant positive correlation between PD for past events and log k in Mandarin speakers, Pearson’s r = 0.51, t_(33)_ = 3.39, *P* = 0.002, n = 35. (**c**) No correlation between PD for past events and log k in English speakers, Pearson’s r = 0.08, t_(37)_ = 0.48, *P* = 0.63, n = 39. Past distances were more strongly correlated with discount rates in Mandarin speakers compared to English speakers (z = 1.98, *P* = 0.050).

We observed a significant 3-way interaction between discount rate, culture, and timeframe (Type III ANOVA; F_(1,~5183.7)_ = 12.80, *P* < 0.001), suggesting that the relationship between log k and event distances differed by timeframe and culture. This prompted us to examine the relationship between discount rate and event distances in each timeframe separately. Overall, participants rated 44.8% of events as occurring in the future and 55.2% of events as occurring in the past. We observed a significant relationship between log k and distance representations of future events (Type III ANOVA; F_(1,~51.6)_ = 4.11, *P* = 0.048), which did not interact with culture (Type III ANOVA; F_(1,~51.6)_ = 1.69, *P* = 0.199). For display purposes, we plotted each participant’s log transformed discount rate against their average PD score for future-rated events (Fig. 6a). The strong positive correlation indicates that individuals who placed future events further from themselves discounted future rewards more steeply (Pearson’s r = 0.30, t_(72)_ = 2.67, two-tailed, *P* = 0.009, 95% CI [0.08, 0.49]). We observed a positive correlation in each culture individually (English: Pearson’s r = 0.21, t_(37)_ = 1.30, two-tailed, *P* = 0.20, 95% CI [− 0.11, 0.49], Mandarin: Pearson’s r = 0.42, t_(33)_ = 2.68, two-tailed, *P* = 0.011, 95% CI [0.10, 0.66]), but in accordance with the model above did not observe a difference between the correlations (Test of difference between two independent correlations, z = 0.98, two-tailed, *P* = 0.33).

When examining past events, we found that the relationship between log k and event distances interacted with culture (Type III ANOVA; F_(1,~52.1)_ = 5.36, *P* = 0.025), which prompted us to examine each culture individually. When looking selectively in Mandarin speakers, we observed a significant relationship between log k and past distance representations (Type III ANOVA; F_(1,~17.0)_ = 6.35, *P* = 0.022). This relationship is visually apparent in Fig. 6b, which depicts Mandarin speakers’ log transformed discount rates plotted against their average distance scores for past-rated events. The strong positive correlation suggests that Mandarin speakers who represented the past as more distant discounted future rewards more strongly (Pearson’s r = 0.51, t_(33)_ = 3.39, two-tailed, *P* = 0.002, 95% CI [0.21, 0.72]). In English speakers, past distance representations were not associated with discount rate (Type III ANOVA; F_(1,~20.0)_ = 1.56, *P* = 0.226); this is supported and visually depicted by a non-significant relationship between English speakers’ log transformed discount rates and their average distance scores for past-rated events (Fig. 6c; Pearson’s r = 0.08, t_(37)_ = 0.48, two-tailed, *P* = 0.63, 95% CI [− 0.24, 0.38]). Univariate statistics further demonstrate that past distance representations were more strongly correlated with discount rates in Mandarin speakers compared to English speakers (Test of difference between two independent correlations, z = 1.98, two-tailed, *P* = 0.050, Cohen’s q = 0.48).

We categorized events as past or future based on the participants’ evaluation of its timeframe. Of the participants’ timeframe ratings, 87.97% aligned with those we assigned and all modeling results replicated when excluding events that were incorrectly characterized by the participants as belonging to the past or future (Supplementary Table S3). In summary, we found that distance representations of the future associated with discount rates across cultures, while distance representations of the past correlated with discounting behaviors selectively in Mandarin speakers.

## Discussion

Mentally representing the past and future is critical for human functioning; doing so allows us to construct a sense of personal identity and prepare for future scenarios^39^. Yet, because time is an immaterial entity, individuals and cultures have developed different ways of conceptualizing, communicating, and imagining it^40^. Through the use of a novel time representation task, we demonstrate that English and Mandarin speakers represent world events differently. English speakers employed largely linear, horizontal schemas, while Mandarin speakers employed less linear schemas and more readily used the vertical dimension when organizing the events. Though our design does not permit causal claims, our findings align with previous work examining the impact of the differential use of horizontal and vertical metaphors in English and Mandarin on speakers’ conceptualizations of time. Explicit approaches in which participants have arranged sequences of cards or pointed to time points in 3D space have demonstrated that Mandarin speakers more readily use the vertical axis when mapping time than English speakers^41,42^. Similarly, spatial priming paradigms in which participants responded to vertically or horizontally arranged temporal sequences have shown that Mandarin speakers more regularly conceptualize time using the vertical dimension^18,19,43^.

In our task, Mandarin speakers’ schemas also involved a horizontal component, which we theorize stems from the strong influence of writing direction on time representation. Traditionally written in vertical columns, Mandarin switched to being written horizontally in 1956 and most newspapers, books, and web materials are now written from left-to-right^44^. Thus, the two dimensional representations we observed may have resulted from the combinatory influence of vertical linguistic metaphors and writing direction. One way to test this would be to examine spatial representations in elderly Mandarin speakers. These individuals would theoretically have more experience with the previous vertically organized writing system. Thus, one might expect them to place fewer events horizontally than our participants, who were between ages 18–30 and born into the horizontal writing era. Altogether, we extend the understanding of the differences in temporal cognition between English and Mandarin speakers to the representation of real world events.

Inherent in all individuals’ representation of time is the closeness of the past and future. According to the construal level theory, distant future outcomes are subject to high-level construal, where focus is placed on the abstract features of the events, and proximal future outcomes are subject to low-level construal, where emphasis is place on the detailed, concrete features^16^. Research in this domain has revealed that inducing a future reward to be construed at a lower level, i.e. enhancing its concreteness with detail, can reduce temporal discounting^4,27–29,31^. We distinguish ourselves from this line of work by measuring the impact of inherent variability in temporal distance representations for world events across individuals.

Irrespective of the space–time schema employed, we observed strong individual variability in the psychological distance with which participants considered the same past and future events. Mandarin speakers represented past and future events farther from the avatar than English speakers, which we theorize results from their more two dimensional use of the canvas space. If the canvas had been elongated along the horizontal axis, English speakers may have shown comparable distances. We also observed substantial variability in discounting behaviors across participants, suggesting that individuals differed in the subjective value that they assigned to the rewards. We found that individuals who represented future world events as further from themselves exhibited less patience for delayed rewards. This effect was not culturally unique, which suggests that distance representations of the future play a fundamental role in temporal decision-making.

The time scale for the time representation task greatly exceeded that of the delay discounting task. Participants were surveyed on world events ranging from the construction of the Pyramids to humans living past 150, while the longest delay presented in the discounting task was 180 days. This indicates that our general conception of a longer, more abstract timescale is invoked when making decisions over much smaller, more concrete timescales. Our time representation task instructed participants to place events relative themselves (the avatar) and we cleared the canvas space after each event placement to minimize the possibility that participants placed events relative to other events. Further investigation is needed, however, to determine if participants would employ similar temporal schemas if allowed to view all events on the canvas at once, where the relationships between the events may play a role in their placements.

Distance representations of past events were associated with discount rates selectively in Mandarin speakers, which we interpret in light of the past focused temporal orientation in Eastern over Western culture. Previous work has shown that East Asians rate past behaviors as more relevant when explaining current events (a theft crime) than North Americans^45^. Further, when describing an assassination, a Chinese-language newspaper more heavily focused on situational factors from the assailant’s past, while an English-language newspaper more strongly emphasized the assailant’s personal attributes^46^. East Asians have been shown to assign greater monetary value to past events over future events, while North Americans assign greater monetary value to the contrary^47^. Lastly, positive ratings of the present-self correlated with well-being in European and Asian Americans, while positive ratings of the past-self correlated with well-being selectively in Asian Americans^48^. This suggests that the past self is more highly valued and integrated with the current self in Eastern culture. Correspondingly, East Asians have reported feeling more continuous with their past selves than North Americans^49^. Altogether, our findings extend the known divergence in temporal orientation between East Asians and North Americans to the temporal decision-making domain.

Of note, we did not observe any statistical differences in the variability of past and future distance representations across or within cultures. English speakers, however, trended towards being less variable in their past distance representations than Mandarin speakers, as shown in Supplementary Table S1. One possibility is that English speakers felt confined in their placement of the past events by the dimensions of the horizontal axis. We believe that this is unlikely because English speakers showed comparable variability to Mandarin speakers in their future distance representations and had the same amount of space along the horizontal axis in the future as in the past. Nevertheless, follow-up studies should explore whether inducing a larger variance in past distance representations in English speakers prompts a relationship between discount rate and past psychological distance.

High delay discounting rates are prevalent in several psychiatric disorders, including addiction^6–8^, obesity^9^, and schizophrenia^10^, which begs the question: do altered representations of time underlie these maladaptive decisions? Indeed, previous work has highlighted the need to explore whether altered perceptions of time contribute to the impulsivity observed in several psychiatric disorders, namely substance abuse and pathological gambling^50,51^. Individuals with anxiety^52^, depression^52^, and post-traumatic stress disorder symptomology^53^ have been shown to abnormally represent the psychological distance of past and future personal events. It remains to be seen, however, whether aberrant conceptualizations extend more generally to individuals’ representations of world events and whether this ultimately influences decision-making. If so, targeting patients’ psychological distance representations through construal level manipulations, for instance by shifting the affective tone or altering the amount of detail associated with the past or future, could offer therapeutic potential.

In conclusion, we demonstrate that English and Mandarin speakers represent historic and futuristic world events using diverging space–time schemas. English speakers employed largely one-dimensional, horizontal schemas to represent past and future events, while Mandarin speakers tended towards two-dimensional, more circular schemas. We observed strong variability in distance representations within each culture, and across cultures individuals who represented future events as more distant exhibited less patience for delayed rewards. Past distance representations were associated with discount rates selectively in Mandarin speakers, which reinforces the notion that cultural temporal focus plays an important role in daily decision-making. Altogether, this work extends the known influence of culture and language on time representation and empirically demonstrates the integral role that temporal psychological distance plays in decision-making.

## Methods

### Participants

44 healthy participants ages 18–30 were recruited from New York, USA and 43 healthy participants from Guangzhou, China. Participants rated their English and Mandarin proficiency (1 = not at all fluent, 5 = completely fluent^43^), and were retroactively excluded if they were fluent (score of 4–5) in the opposing language or if they were not fluent in the language of their own country (score of 1–2). Three English speakers were excluded for high Mandarin proficiency and one English speaker was excluded for an inability to follow task instructions. Three Mandarin speakers spoke a local dialect fluently and were excluded for low Mandarin proficiency. Forty English speakers (M = 23.5 years, SD = 2.75, n = 22 female) and 40 Mandarin speakers remained (M = 23.0 years, SD = 3.05, n = 20 female). No difference in average age was observed across the cultures (Wilcoxon sum rank test; W = 919.5, two-tailed, *P* = 0.25, 95% CI for the difference in location [− 1.00, 2.00]).

All participants provided written informed consent and were financially compensated for their participation. English speakers were paid $15 in cash for completing the tasks on the day of the experiment and were paid their choice after a specified delay on a randomly selected trial from the delay discounting task. Mandarin speakers were paid their choice on a randomly selected trial from the discounting task after the delay specified, and were reimbursed for transportation to the lab. The Institutional Review Board at the Icahn School of Medicine at Mount Sinai and the ethical committee of South China Normal University approved this experiment. Compliance with all relevant ethical regulations was ensured throughout the study and methods were carried out in accordance with relevant guidelines and regulations.

### Procedures and materials

Participants completed two tasks, a time representation task and a delay discounting task, and five surveys. Surveys were completed in the following order for all participants: event evaluation, demographic, self-continuity, task rationale, and state-trait anxiety inventory (STAI). Experiment order for the time representation and delay discounting tasks was counterbalanced in each study sample. All materials were developed in English and translated to Mandarin by two bilingual researchers at South China Normal University (for translated task instructions, see Supplementary Table S4). English speakers completed the tasks on a Windows Lenovo ThinkPad (15.5in), and Mandarin speakers on either an Acer Aspire e15 (15.6in), Acer V193W (19in), or Acer G195WVAb (19in).

### Time representation task

Participants viewed a 700pt. × 700pt. blank white canvas on a computer screen. At the top of the canvas an event was displayed in a 50pt. box. At the center of the canvas was a 60pt. × 120pt. blue avatar. The blue avatar was chosen to avoid visually apparent racial indicators. Overlaid on the stomach of the avatar was a yellow circle with a 10.5pt .radius. Instructions shown in Supplementary Table S4 remained on the screen at all times. Participants were verbally instructed to assume that all events have happened or could happen, meaning they were instructed to assume that all events were realistic. Participants picked up the yellow dot with the mouse and placed it anywhere within the 700pt. × 700pt. canvas using a drag-and-drop approach. Grid coordinates were not apparent to the participant, but were recorded in the background for the final placement of the event.

Participants had unlimited time to answer and each time a new event appeared the yellow dot reappeared on the stomach (center) of the avatar. Participants were not allowed to go back and change the location of previous placements. Events appeared serially and randomly on the screen. The canvas refreshed after each placement to encourage participants to place events based on the represented distance of the event from themselves (the avatar), rather than the relative distance of events to each other. Participants rated 6 events in a practice round before completing the full 72-event task. This training served to initialize all participants to the space using the same set of events. It also sought to familiarize participants with the timespan over which events would be surveyed, such that they would have a mental representation of time on the canvas when beginning the task.

We developed four versions of the task to match the sex of the avatar with the sex of the participant and to counterbalance the direction that the avatar was facing. In versions 1 and 2 a female avatar was facing to the right and left respectively (from the perspective of the participant viewing the screen). In version 3 and 4 a male avatar was facing to the right and left respectively. Our inclusion of left and right facing avatars stemmed from the literatures’ indication that linguistic front/back references to the past and future may be flipped in English and Mandarin, whereby the past is referred to as behind in English and in front in Mandarin^21^. Qualitatively, we did not find strong evidence for participants placing events in line with this mapping. Participants largely aligned with writing direction, placing the past on the left and future on the right regardless of the avatar’s direction. Participants were counterbalanced across versions, whereby 20 English and 20 Mandarin speakers completed the task with the avatar facing to the right and 20 English and 20 Mandarin speakers completed the task with the avatar facing to the left. We did not observe any differences in linearity by task version (Supplementary Fig. S5, Kruskal–Wallis rank sum test; English: χ^2^_(3)_ = 2.35, *P* = 0.50, Mandarin: χ^2^_(3)_ = 1.21, *P* = 0.75) or psychological distance by task version (Supplementary Fig. S5, Kruskal–Wallis rank sum test; English: χ^2^_(3)_ = 2.87, *P* = 0.41, Mandarin: χ^2^_(3)_ = 3.93, *P* = 0.27). This task was developed in JavaScript.

### Stimuli

Events were selected using a pre-task survey in an independent sample of 30 English speakers in the USA (M = 21.0 years, SD = 2.75, n = 17 female) and 30 Mandarin speakers in China ages 18–30 (M = 20.6 years, SD = 2.58, n = 15 female). No difference in average age was observed across the cultures (Wilcoxon sum rank test, W = 485.5, two-tailed, *P* = 0.60, 95% CI for the difference in location [− 1.00, 2.00]). Individuals were sent an online survey with 184 internationally known events and were asked to rate the events on arousal [0 = not exciting-7 = very exciting], familiarity [0 = not familiar-7 = very familiar], valence [0 = negative-7 = positive], using a sliding scale and date (when did/will this happen?) [further past, closer past, closer future, further future, and never] via a multiple-choice response. Within each culture, mean arousal, valence, and familiarity ratings were calculated for each event by averaging scores across the 30 participants. Results were then filtered to remove events that participants were not familiar with (avg. familiarity score of 1.5 and below) or events that were considered too unrealistic (10 or more participants selected “never” for the date rating). This filtering occurred in each culture individually and results were merged to examine which events remained in both cultures.

Thirty-six past and 36 future events were selected and non-parametric two-tailed Wilcoxon sum rank tests verified that there were no differences in arousal, familiarity, or valence for past events (Supplementary Fig. S6, arousal: W = 697.5, *P* = 0.58, familiarity: W = 802.5, *P* = 0.08, valence: W = 636.5, *P* = 0.90) or for future events between the cultures (Supplementary Fig. S6, arousal: W = 713.5, *P* = 0.46, familiarity: W = 487, *P* = 0.07, valence: W = 507, *P* = 0.11). See Supplementary Table S5 for a complete list of training events and their Mandarin translations and Supplementary Table S6 for a complete list of task events and their Mandarin translations. In the current sample, English speakers were significantly more aroused by and familiar with the selected events (Supplementary Fig. S6, Wilcoxon sum rank test, two-tailed, arousal: W = 1,095.5, *P* = 0.005, familiarity: W = 1,154.5, *P* < 0.001). No difference was observed in valence ratings (Wilcoxon sum rank test, W = 802.5, two-tailed, *P* = 0.98). English speakers were significantly more educated than Mandarin speakers (Pearson’s chi-squared test, χ^2^_(2)_ = 26.23, *P* < 0.001), however, education level was not related to the familiarity for the events in either culture (English: Spearman’s rho = 0.08, S = 9,820.3, two-tailed, *P* = 0.63, Mandarin: Spearman’s rho = 0.27, S = 7,764, two-tailed, *P* = 0.09).

### Delay discounting task

Participants viewed 51 choices and selected whether they would like a smaller amount of money now or a larger amount of money after a variable delay (task used in^33^). The English task displayed amounts in USD ($) and the Mandarin version in CNY (¥). For English speakers, the now options ranged from $10 to $34 and the delay amounts were either $25, $30, or $35. For Mandarin speakers, the now options ranged from ¥33.50 to ¥113.90 and the delay amounts were either ¥83.75, ¥100.50, or ¥117.25. To convert the currencies, we examined the purchasing power in each country by comparing the amount of time an individual would have to work in New York and Beijing to earn a Big Mac. According to the UBS in 2018, an individual would have to work 51.0 m in Beijing and 15.2 m in New York, leading to a conversion of 1 USD = 3.35 CNY (https://www.ubs.com/minisites/prices-earnings/en/cities/beijing/). The delays varied from 1 to 180 days and each participant was given an unlimited amount of time to respond. After making a response, a white check mark appeared on the screen for 500 ms to indicate which answer was just chosen, followed by a 1000 ms inter-trial interval, and the subsequent question. Now and later options appeared randomly on either the right or left side of the screen.

To encourage realistic decisions, participants were informed that they would be paid their response on a randomly selected trial before completing the task. To explain this concept, English speakers were verbally given the example (Mandarin speakers the translated equivalent) that if they selected $25 in 30 days as one of their answers, this would be placed into a lottery with all of their other responses. One response would be randomly selected and if this was chosen, they would be mailed a check for $25 after 30 days. After this example the experimenters again emphasized that the payment and delay would be based on a randomly selected trial from their responses. The task was programmed using E-Prime 2.0 (https://pstnet.com).

### Task surveys

Participants completed an event evaluation survey where they evaluated the 72 time representation events on arousal, familiarity, valence, and date. The scales mirrored those of the pre-task survey, however, since participants were told to assume all events were realistic, the date question no longer included a never option. The event evaluation survey was administered to English speakers via SurveyMonkey (https://www.surveymonkey.com) and Mandarin speakers via Wenjuanwang (https://www.wenjuan.com). Participants completed a demographic survey where they self-reported their age, sex, race, language fluency, education, occupation, income, socioeconomic status, financial security, and religious or spiritual views. On the task rationale survey, participants explained their decisions in the discounting task, their strategy for the time representation task, and sketched their time representation schemas (data not included). On the self-continuity survey, participants reported how similar they felt to their past self, 10 years ago, and future self, 10 years from now (survey used in^54^, see Supplementary Fig. S7 for results). Lastly, participants completed the standard STAI, which consisted of 40 questions examining their state and trait stress (see Supplementary Fig. S7 for results). We did not observe any significant correlations between state stress, trait stress, future self-continuity, and past self-continuity and psychological distance or discount rate in either English or Mandarin speakers (Supplementary Table S7). These surveys were administered to English speakers using REDCap (https://www.project-redcap.org) and Mandarin speakers using Wenjuanxing (https://www.wjx.cn).

### Analysis methods

All analyses were conducted in R v. 3.6.0, except for the discount rate estimation, which was run in MATLAB R2015a (The MathWorks, Inc.). Statistics were computed using non-parametric Wilcoxon sum rank, Wilcoxon signed rank, and Kruskal Wallis tests, parametric Two Sample *t*-tests, Pearson correlations, and Spearman correlations as indicated in the text. All tests were two-tailed. A Cohen’s d effect size was used when comparing linearity across cultures and was calculated by dividing the mean difference by the pooled standard deviation. A Cohen’s q effect size was calculated for the comparison of correlations across English and Mandarin speakers^55^. An eta-squared (*η*^2^) effect size was calculated for the linearity ANOVA using the eta_sq function from the sjstats package in R^56^.

We quantified linearity by fitting a linear model, lm(Y coordinate ~ X coordinate) to each participant’s event placements and extracting the residual standard deviation associated with the model. We computed event distances by calculating the length of the vector connecting each event placement to the center of the canvas (stomach of the avatar) and PD by averaging the vector lengths across all event placements for each participant. We then dissected this PD value into the average PD for past-rated events and future-rated events in each participant. In an exploratory analysis, we examined whether the difference in PD between “further” and “closer” past/future events was associated with discount rate, however, we did not find evidence for a relationship (Supplementary Table S8).

Discount rates were estimated using a logistic regression model in MATLAB, where participants’ choices were fit using maximum likelihood estimation^34^. The subjective values of options were estimated using a hyperbolic discounting model: SV = A/(1 + kD). A represented the amount of the option, SV the subjective value of the option, D the delay, and k represented an individual discount parameter. Analyses were conducted using scripts provided by the Kable Laboratory.

The linearity multiple linear regression model was built using the lm() function in R and served to ensure that the effect remained when controlling for demographic factors. The regressions estimated the Type III Sum of Squares. Regressors and their scales for the linearity model included: mean event arousal, familiarity, and valence ratings (1–7), age (18–30), sex (male, female), education (high school, college, post graduate), and culture (English, Mandarin). Linear mixed models were built to examine the relationship between event distances and discount rates. These models were built using the lme4 and lmerTest packages in R and estimated significance using the Type III Sum of Squares. Fixed effects and their scales included: culturally adjusted income bracket (1–7, decline to respond, I don’t know), socioeconomic status (1–10), financial security (1–5, decline to respond), age (18–30), sex (male, female), education (high school, college, post graduate), culture (English, Mandarin), timeframe (past, future), and log k. Event name (72 total events) and participant (74 total participants) were included as random effects. In the models above, event ratings, age, SES, psychological distance, and log k were coded as continuous variables. Sex, education, culture, income bracket, financial security, and timeframe were coded as factor variables.

## Supplementary information

Supplementary Information.

## Data Availability

Data used to support the conclusions of this study is available online at: https://osf.io/kaqe9/.

## Code availability

A tutorial for calculating linearity and psychological distance is available online at: https://osf.io/kaqe9/.
