# Supplementary material for: Delay discounting decisions are linked to temporal distance representations of world events across cultures
Source: Sci Rep. 2020 Jul 31;10:12913. doi: 10.1038/s41598-020-69700-w (PMC7395128; doi:10.1038/s41598-020-69700-w)
Supplement: Supplementary file 1 — Supplementary Information. [file 41598_2020_69700_MOESM1_ESM.pdf]

Supplemental Materials

Delay discounting decisions are linked to temporal distance representations of world events across cultures.

Denise E. Croote[1,2], Baojun Lai[3], Jingchu Hu[3], Mark G. Baxter[1], Alison Montagrin[1,2,4], & Daniela Schiller[1,2,4]

[1]The Nash Family Department of Neuroscience, Icahn School of Medicine at Mount Sinai, New York, NY 10029, USA
[2]Department of Psychiatry, Icahn School of Medicine at Mount Sinai, New York, NY 10029, USA
[3]School of Psychology and Center for Studies of Psychological Application, South China Normal University, Guangzhou 510631, China
[4]These authors contributed equally: Alison Montagrin and Daniela Schiller

Corresponding Authors
Daniela Schiller
Icahn School of Medicine at Mount Sinai, New York, NY 10029, USA
Email: daniela.schiller@mssm.edu
Tel: 212-824-8977

Alison Montagrin
University of Geneva, Geneva 1202, Switzerland
Email: alison.montagrin@unige.ch
Tel: +41 22 379 07 51

Keywords: delay discounting; psychological distance; cross-cultural; decision-making

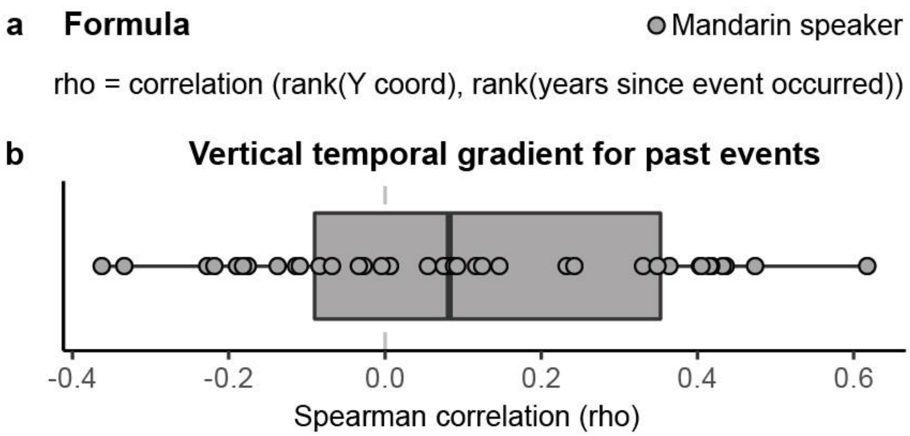

Supplementary Figure 1: Examination of vertical event placements in Mandarin speakers. Linguistically, *shàng* (up, top) refers to events earlier in time and *xià* (down, bottom) events later in time. As a result, we would expect more distant past events to be placed above more recent past events. **(a)** We calculated a rank correlation between the true temporal distances of past events and the Y coordinates of the event placements in each participant. **(b)** Across Mandarin speakers, we observed a weak, but significant positive correlation, indicating that participants tended to place more distant past events higher up on the canvas than more recent past events (Spearman's rho=0.10, One Sample t-test; $t_{(39)}$=2.61, two-tailed, P=0.01, 95% CI [0.02, 0.18]). We included only events that Mandarin speakers correctly identified as occurring in the past in this analysis (Mdn=34, range=20-36). We did not pursue a similar analysis for the future, as we are not able to obtain precise estimates of the true temporal distances of future events.

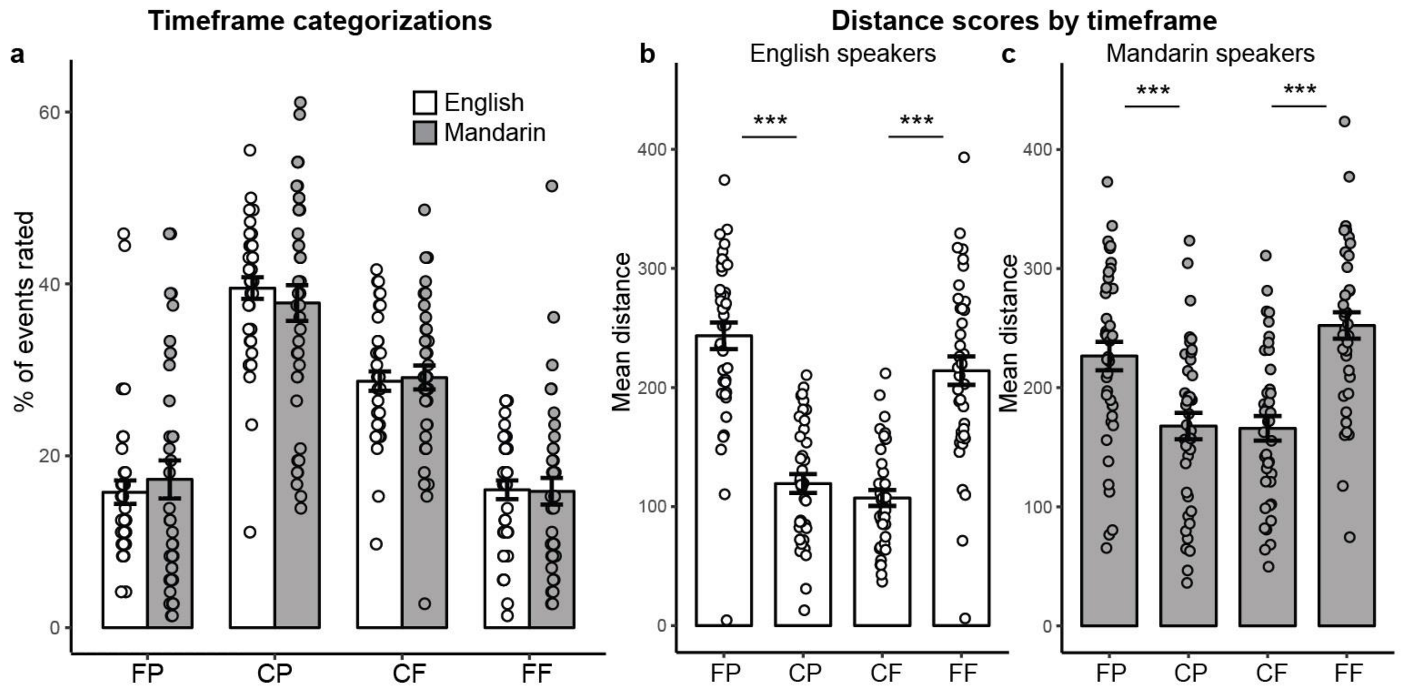

Supplementary Figure 2: Timeframe ratings and distance representations by timeframe. (a) There was no difference in the percentage of events rated as occurring in each timeframe by culture (Wilcoxon sum rank test, two-tailed, further past (FP): W=861.5, P=0.56, closer past (CP): W=841, P=0.70, closer future (CF): W=765, P=0.74, further future (FF): W=875.5, P=0.47. **(b)** English speakers placed further past-rated events significantly farther from the avatar than closer past events (FP PD: M=243.37, SEM=11.10, CP PD: M=119.32, SEM=7.90, Wilcoxon signed rank test, V=815, two-tailed, P<0.001) and further future-rated events significantly farther from the avatar than closer future events (FF PD: M=214.08, SEM=11.93, CF PD: M=107.24, SEM=6.65, Wilcoxon signed rank test, V=812, two-tailed, P<0.001). **(c)** Mandarin speakers placed further past rated-events significantly farther than closer past events (FP PD: M=226.54, SEM=12.00, CP PD: M=167.82, SEM=11.02, Wilcoxon signed rank test, V=760, two-tailed, P<0.001) and further future-rated events significantly farther than closer future events (FF PD: M=252.22, SEM=11.07, CF PD: M=165.91, SEM=10.28, Wilcoxon signed rank test, V=819, two-tailed, P<0.001). Error bars = SEM. *** P<0.001.

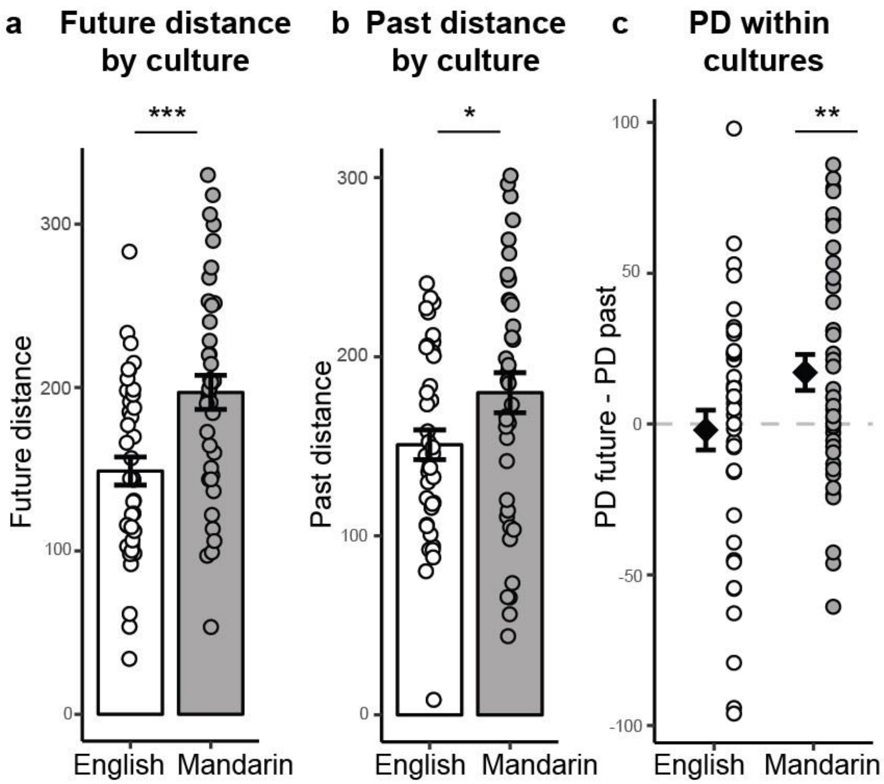

Supplementary Figure 3: Past and future PD representations. (a) Mandarin speakers represented future-rated events as further from themselves than English speakers (Mandarin: M=196.96, SEM=10.39, English: M=148.84, SEM=8.61, Two Sample t-test; $t_{(78)}$=3.56, two-tailed, P<0.001, 95% CI for the difference in means [21.25, 75.00]). **(b)** Mandarin speakers represented past-rated events as further from themselves than English speakers (Mandarin: M=179.88, SEM=11.23, English: M=150.83, SEM=8.26, Two Sample t-test; $t_{(78)}$=2.08, two-tailed, P=0.040, 95% CI for the difference in means [1.31, 56.80]). **(c)** Within cultures, Mandarin speakers placed past-rated events significantly closer to themselves than future-rated events (Mean of the difference=17.08, SEM=5.94, Paired t-test; $t_{(39)}$=2.87, two-tailed, P=0.007, 95% CI for the difference in means [5.05, 29.10]). Alternatively, English speakers did not differ in the average PD that they placed past and future-rated events (Mean of the difference=-1.99, SEM=6.60, Paired t-test; $t_{(39)}$=-0.30, two-tailed, P=0.764, 95% CI for the mean of the differences [-15.34, 11.35]). Error bars = SEM. *P<0.05, **P<0.01, ***P<0.001.

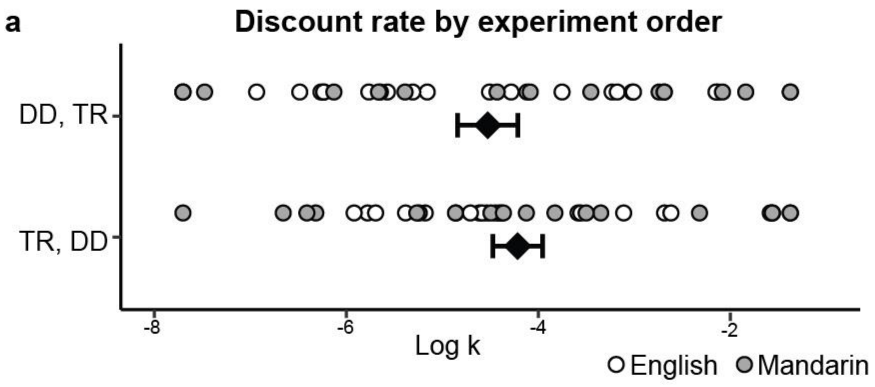

Supplementary Figure 4: Experiment order validation. Several empirical studies have demonstrated that cueing episodic future thinking, in particular cueing individuals to imagine positive future scenarios, prior to intertemporal choice tasks can reduce temporal discounting (see Bulley, Henry & Suddendorf 2016 for a review). **(a)** We did not observe a difference in discount rate by experiment order, i.e. whether participants completed the time representation (TR) task first and delay discounting (DD) task second or the converse (Two Sample t-test; $t_{(72)}=0.77$, two-tailed, $P=0.45$, 95% CI for the difference in means [-0.50, 1.13]). The tasks described in the body of literature above cued episodic future thinking with the simulation of highly personal future occurrences. It is possible that we did not observe an effect because our task involved past and future world events, rather than personal future events.

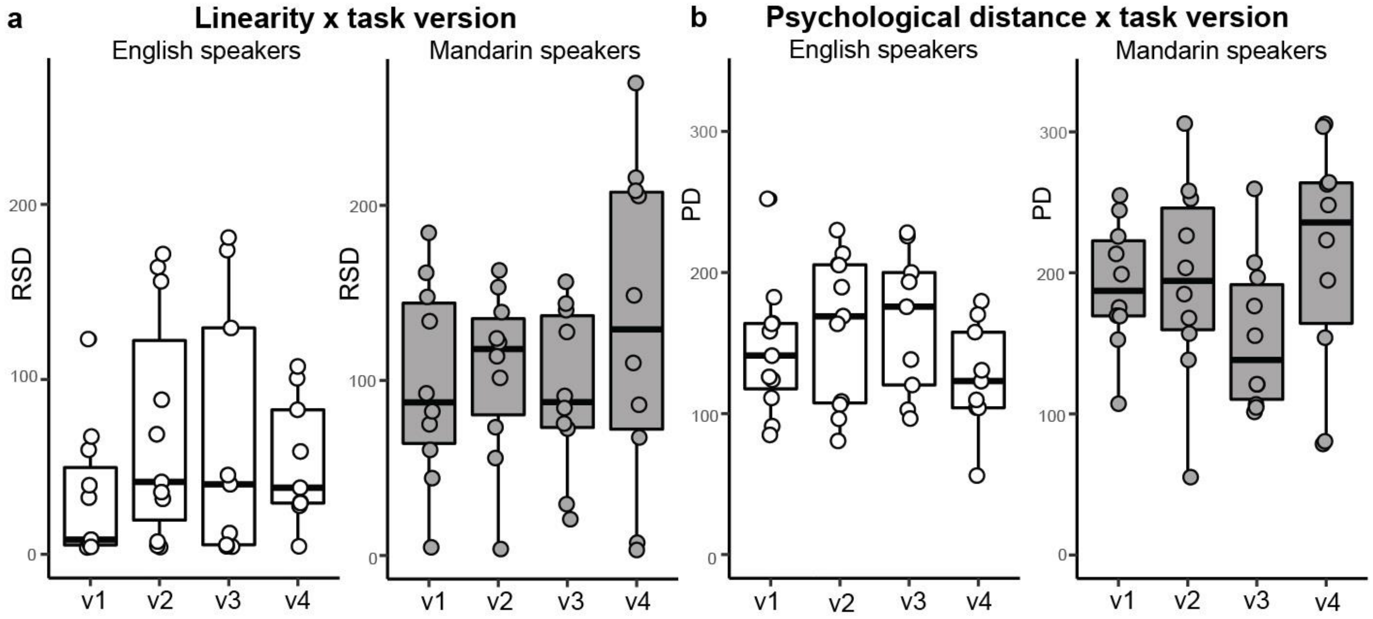

Supplementary Figure 5: Time representation version validations. We developed four versions of the task to match the sex of the avatar with the sex of the participant and to counterbalance the direction that the avatar was facing**. (a)** There was no difference in linearity by time representation task version (Kruskal-Wallis rank sum test, English: $\chi^2_{(3)}$=2.35, P=0.50, Mandarin: $\chi^2_{(3)}$=1.21, P=0.75) or **(b)** psychological distance by time representation task version (Kruskal-Wallis rank sum test, English: $\chi^2_{(3)}$=2.87, P=0.41, Mandarin: $\chi^2_{(3)}$=3.93, P=0.27). Error bars=SEM. Box plots; center line=median, box limits=Q1 and Q3, whiskers=smallest/largest value no further than 1.5x IQR.

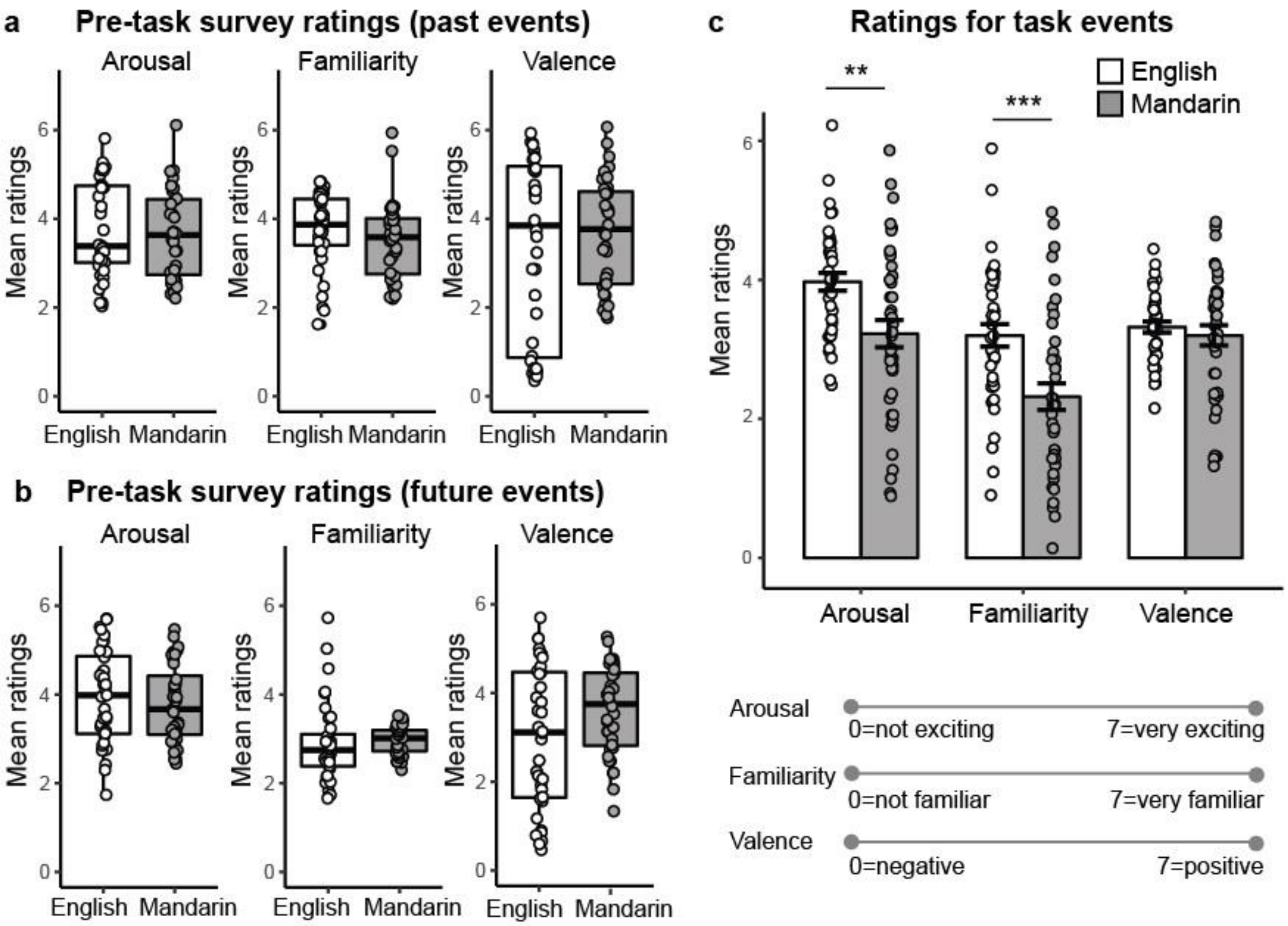

Supplementary Figure 6: Arousal, familiarity, and valence event ratings. Events were selected using a pre-task survey in an independent sample of 30 English and 30 Mandarin speakers. Individuals rated 184 events on arousal, familiarity, and valence (scale =1-7). Results were filtered to remove events that were rated as too unfamiliar or unrealistic. Thirty-six past and 36 future events were selected. **(a)** There were no differences in arousal, familiarity, or valence for the selected past events (Wilcoxon sum rank test, a: W=697.5, P=0.58, f: W=802.5, P=0.08, v: W=636.5, P=0.90) or **(b)** future events (Wilcoxon sum rank test, a: W=713.5, P=0.46, f: W=487, P=0.07, v: W=507, P=0.11) between the cultures. **(c)** In the current sample, English speakers were significantly more aroused by and familiar with the events than Mandarin speakers (Wilcoxon sum rank test, a: W=1095.5, P=0.005, f: W=1154.5, P<0.001, v: W=802.5, P=0.98. Error bars=SEM. **P<0.01, ***P<0.001. Box plots; center line=median, box limits=Q1 and Q3, whiskers=smallest/largest value no further than 1.5x IQR.

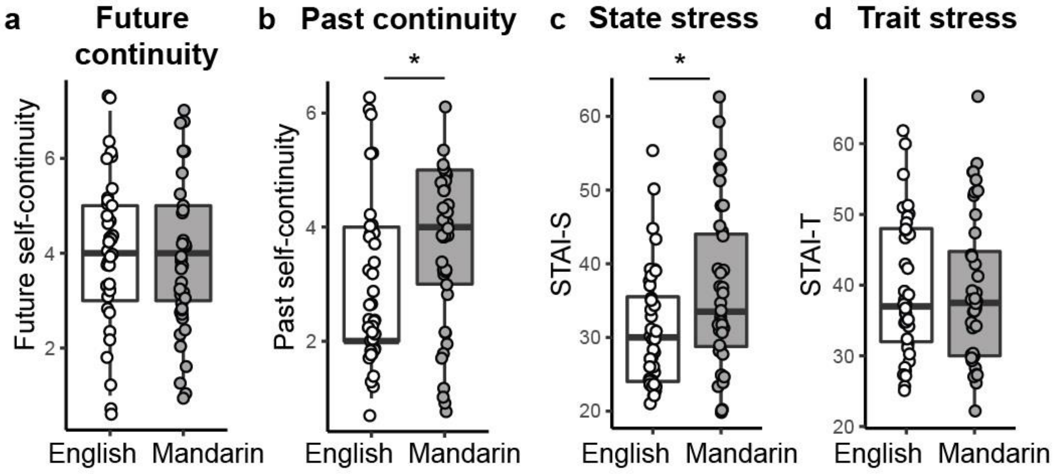

Supplementary Figure 7: Self-reported continuity and stress by culture (a) We did not observe a difference in future self-continuity between the cultures (Mandarin: M=3.80, SEM=0.25, English: M=4.05, SEM=0.24, Wilcoxon sum rank test: W=898.5, two-tailed, P=0.34). **(b)** Mandarin speakers felt more continuous with their past selves than English speakers felt with their past selves (Mandarin: M=3.58, SEM=0.21, English: M=2.93, SEM=0.22, Wilcoxon sum rank test: W=571.5, two-tailed, P=0.025). **(c)** Mandarin speakers reported being more state stressed than English speakers (Mandarin: M=36.20, SEM=1.75, English: M=31.20, SEM=1.26, Wilcoxon sum rank test: W=577.5, two-tailed, P=0.032). **(d)** We did not observe a difference in trait stress between the cultures (Mandarin: M=39.38, SEM=1.67, English: M=39.70, SEM=1.54, Wilcoxon sum rank test: W=811.5, two-tailed, P=0.916). Error bars=SEM. *P<0.05. Box plots; center line=median, box limits=Q1 and Q3, whiskers=smallest/largest value no further than 1.5x IQR.

Within cultures	
English speakers: future PD vs. past PD	F test to compare two variances; $F_{(39,39)}$=1.09, P=0.794, 95% CI [0.58, 2.06]
Mandarin speakers: future PD vs. past PD	F test to compare two variances; $F_{(39,39)}$=0.86, P=0.633, 95% CI [0.45, 1.62]
Across cultures	
Past PD in English vs. Mandarin speakers	F test to compare two variances; $F_{(39,39)}$=0.54, P=0.059, 95% CI [0.29, 1.02]
Future PD in English vs. Mandarin speakers	F test to compare two variances; $F_{(39,39)}$=0.69, P=0.244, 95% CI [0.36, 1.30]
Discount rate in English vs. Mandarin speakers	F test to compare two variances; $F_{(38,34)}$=0.55, P=0.074, 95% CI [0.28, 1.06]

Supplementary Table 1: Comparisons of task variability within and across cultures. We did not observe a difference in the variability of past and future distance representations across cultures, nor did we observe a difference in the variability of past and future distance scores within each culture. Past and future PD were strongly correlated within each culture. The strength of the correlations approached, but did not statistically differ across cultures (Mandarin: Pearson's r=0.86, English: Pearson's r=0.70, Test of difference between two independent correlations, z=1.73, two-tailed, P=0.08). Lastly, we did not observe a difference in the variability in discount rates across cultures.

Model	Description	Formula
1	All event distances vs. log k across cultures	event distances ~ income bracket + SES + financial security + age + sex + education + log k*timeframe*culture + (1 | event name) + (1 | subject id)
2	Future event distances vs. log k across cultures	future event distances ~ income bracket + SES + financial security + age + sex + education + log k*culture + (1 | event name) + (1 | subject id)
3	Past event distances vs. log k across cultures	past event distances ~ income bracket + SES + financial security + age + sex + education + log k*culture + (1 | event name) + (1 | subject id)
4	Past event distances vs. log k in Mandarin speakers	past event distances ~ income bracket + SES + financial security + age + sex + education + log k + (1 | event name) + (1 | subject id)
5	Past event distances vs. log k in English speakers	past event distances ~ income bracket + SES + financial security + age + sex + education + log k + (1 | event name) + (1 | subject id)

Supplementary Table 2: Linear mixed models. The dependent variable, event distances, represents the length of the vectors connecting each event placement to the center of the canvas. This is a single value for each event. The predictor, log k, represents the individual discount rate for each participant. The fixed effects of SES (1-10), age (18-30), and log k were coded as continuous variables, while income bracket (1-7, decline to respond, I don't know), financial security (1-5, decline to respond), sex (male, female), education (high school, college, post graduate), timeframe (past, future), and culture (English, Mandarin) were coded as factor variables. The random effect, event name, included 72 total events and subject id included 74 participants for models 1-3, 35 participants for model 4, and 39 participants for model 5.

Model	Description	Result
1	All event distances vs. log k across cultures	Significant 3-way interaction between discounting rate, culture, and timeframe (Type III ANOVA; $F_{(1,\sim 4541.8)}=17.51$, $P<0.001$).
2	Future event distances vs. log k across cultures	Significant relationship between log k and future event distances (Type III ANOVA; $F_{(1,\sim 51.6)}=4.23$, $P=0.045$), which did not interact with culture (Type III ANOVA; $F_{(1,\sim 51.7)}=1.31$, $P=0.257$).
3	Past event distances vs. log k across cultures	Relationship between log k and past event distances interacted with culture (Type III ANOVA; $F_{(1,\sim 51.9)}=5.66$, $P=0.021$).
4	Past event distances vs. log k in Mandarin speakers	Significant relationship between log k and past event distances in Mandarin speakers (Type III ANOVA; $F_{(1,\sim 17.0)}=6.71$, $P=0.019$).
5	Past event distances vs. log k in English speakers	Relationship between log k and past event distances was not significant in English speakers (Type III ANOVA; $F_{(1,\sim 20.0)}=1.55$, $P=0.227$).

Supplementary Table 3: Linear mixed model results replicated with event exclusions. Following the time representation task, participants rated the date range of each event (further past, closer past, closer future, or further future) and events were categorized as past or future based on the individual participants' evaluation of its timeframe in all analyses described in the manuscript. Of the participants' timeframe ratings, 87.97% aligned with those assigned by the experimenters and listed in Supplementary Table 6 below (English = 90.95%, Mandarin = 84.64%). After excluding the events that were incorrectly characterized as belonging to the past or future, the conclusions drawn from the models with all events replicated (n=39 English, n=35 Mandarin).

Task	English instructions	Mandarin instructions
Time representation	On the screen above you will see an event appear. Please think about when this event has occurred or when it could occur. Then, imagine that you are the avatar in the canvas above. Place the yellow dot anywhere on the canvas to reflect when you feel the event occurs in relation to yourself.	你将看到屏幕上方出现一个事件，请思考这个事件发生的时间，这可能是发生在过去的事情，也可能在未来发生。 然后，想象屏幕里的人像就是你自己。 你可以移动黄色圆点到屏幕上的任何地方，直到你感觉黄色圆点的位置可以表示这个事件的发生时间。
Delay discounting	The goal of this experiment is to understand how you make choices about money. You will read these instructions and then answer 51 questions. For each question, you will be asked to make a choice between two options. It is very important that you understand these instructions. If you have questions, please ask before the experiment begins. Each question will ask if you want money now or in the future. You will see two options. In the example below, the option on the left is the amount of money you could get today. The option on the right is the amount of money you could get in the future. You have to decide if you want $9 now (today) OR $30 in 102 days. To make your choice, you will press a button. If you want the option on the left, press the BLUE button. If you want the option on the right, press the YELLOW button. The amounts and delays will be different for each question, and the now and future options may switch sides, so you should look at each option carefully. There are no right or wrong answers. You only need to tell us which amount of money you would rather have. After you push the button, a checkmark will pop up, showing which side you pressed. For example, the checkmark below would appear if you chose the option on the right. Are these choices real? After you finish answering the questions, one question from this task will be randomly selected. We will pay you the amount you chose on this question after the delay period that you selected. It is very important that you understand the instructions. If you have questions, please ask us.	这个实验的目的是了解你如何在金钱上做出决策。请你阅读指导语，然后回答 51 个问题。每个问题要求你在两个选项中做出选择。请仔细阅读指导语，如果有任何问题，请在实验开始前询问。每个问题都会问你是想要现在还是将来得到钱。你将看到两个选项。在下面的例子中，左边的选项是你今天能得到的钱。右边的选项是你将来可以得到的钱。你必须决定是现在(今天)要 30.15 元，还是 102 天后要 100.50 元。你需要按一个按钮来做出选择。如果你想要左边的选项，按蓝色按钮。如果你想要右边的选项，按黄色按钮。每个问题的数量和延迟会有所不同，现在和未来的选项可能会有不同，所以你需要仔细地查看每个选项。没有正确或错误的答案。你只要按键告诉我们你想要多少钱就行了。按下按钮后，会弹出一个复选框，显示你按的是哪边。例如，如果您选择右边的选项，下面的复选框将会出现：回答完问题后，我们将从这个任务中随机选取一个题目。我们将在你选择的延迟期后支付您在这个题目上选择的金额。请理解以上指导语。如果你有任何问题，请询问我们。

Supplementary Table 4: Task instructions. The time representation instructions remained on the screen at all times. The delay discounting instructions were shown in advance of the task.

Past events		Future events	
Columbus reaches the new world	哥伦布到达新世界	Humans live underground	人类可以在地下生活
Death of Princess Diana	英国戴安娜王妃死亡	The sun explodes	太阳爆炸
First harry potter movie	第一部哈利波特电影发行	People select the gender of their children	人们可以选择孩子性别

Supplementary Table 5: Time representation training events. Three past and 3 future events were presented during the time representation training. This training served to initialize all participants to the space using the same set of events. It also sought to familiarize participants with the timespan over which events would be surveyed, such that they would have a mental representation of time on the canvas when beginning the task.

Past events		Future events	
Britain Secedes from the European Union	英国脱离欧盟	Artificial humans	人造人产生
Construction of the Pyramids	埃及建成金字塔	Automated driving	自动驾驶技术普及
Creation of the European Union	欧盟成立	Coal supplies exhausted	煤炭耗尽
Creation of the Printing Press	创造印刷机	Division of Pakistan	巴印战争
Death of Marilyn Monroe	玛丽莲·梦露自杀	Evidence that Dark Matter exists	暗物质存在的证据
Death of Nelson Mandela	南非总统纳尔逊·曼德拉的死亡	Flying cars used by the public	大众使用飞行汽车
Death of V.I. Lenin	列宁去世	Gasoline cars extinct	汽油汽车消失
Disintegration of the Soviet Union	苏联解体	Global warming 10 degrees	全球变暖 10 度
Dolly the sheep cloned	克隆多利羊	Growth of a new radical faction	美国产生第三个流派
Earthquake in Haiti	伤亡巨大的海地地震	Holograms used by the public	全息图片被公众使用
Earthquake in Japan	伤亡重大的日本地震	Human cloning	人类克隆
Einstein Theory of Relativity	爱因斯坦相对论	Humans live past 150	人类活过 150 岁
Establishment of the Space Station	第一个宇宙空间站的建立	Humans settle N + S Poles	人类在北极和南极居住
European Reformation	欧洲改革	Humans vacation on Mars	人类在火星上度假
Fall of the Berlin Wall	推翻柏林墙	Humans wear permanent info-keeping devices	人类佩戴永久性信息设备
First Automobile	第一辆汽车	Impeachment of Donald Trump	美国总统唐纳德·特朗普被弹劾
First Industrial Revolution	第一次工业革命	Last person from the 1900s dead	20 世纪最后一个人死亡
First Olympic winter games	第一届冬奥会	New American President	美国有新的总统
First portable phone call	第一部移动手机	New healthcare system in U.S.	美国医疗保健系统革新
First Star Wars film released	星球大战题材电影上映	New international pop star	出现新国际流行歌手
Founding of China	中国成立	New medical epidemic	新的医疗方法流行
Founding of Google	谷歌建立	New social media platform	产生新的社交媒体平台
Hitler becomes German Chancellor	希特勒成为德国总理	Nuclear energy recycled	核能循环利用
JFK Assassinated	肯尼迪被暗杀	Nuclear fission	发生核裂变
Michael Jordan retires	迈克尔·乔丹从 NBA 退役	Oceans begin to be farmed	海洋开始种植
Nazi Rule in Europe	纳粹统治在欧洲	Post offices become obsolete	邮局消失
Newton discovers gravity	牛顿发现万有引力	Radiation spreads from the Pacific	太平洋扩大
Reuniting of East and West Germany	德国合并	Robots develop self awareness	机器人有自我意识
The Berlin Airlift begins	柏林空运开始	Run out of petroleum	石油耗尽
The Boxer Rebellion	义和团运动	The Dead Sea dries	死海消失
The Bubonic Plague	黑死病	The development of self-healing concrete	混凝土自动粘合
Treaty of Versailles ends WW1	凡尔赛条约终止一战	The melting of Greenland	格林兰岛融化
Vietnam War	越南战争	Tracking technology embedded in humans	使用人类追踪技术
World War I	一战	Travel to space by plane	人类乘飞机到太空旅行
World's first telephone call	第一个电话	Universal language	世界使用通用语言
Zika Outbreak	国际寨卡病毒爆发	World population rises to 10B	世界人口 100 亿

Supplementary Table 6: Time representation events. Thirty-six past and 36 future events were presented during the time representation task.

	State stress vs. PD	Trait stress vs. PD
English speakers	Pearson's r=0.16, $t_{(38)}$=0.97, two-tailed, P=0.34, 95% CI [-0.16, 0.45]	Pearson's r=-0.25, $t_{(38)}$=-1.58, two-tailed, P=0.12, 95% CI [-0.52, 0.07]
Mandarin speakers	Pearson's r=0.18, $t_{(38)}$=1.13, two-tailed, P=0.27, 95% CI [-0.14, 0.47]	Pearson's r=0.18, $t_{(38)}$=1.10, two-tailed, P=0.28, 95% CI [-0.14, 0.46]
	State stress vs. Log k	Trait stress vs. Log k
English speakers	Pearson's r=0.24, $t_{(37)}$=1.48, two-tailed, P=0.15, 95% CI [-0.09, 0.51]	Pearson's r=-0.08, $t_{(37)}$=-0.51, two-tailed, P=0.61, 95% CI [-0.39, 0.24]
Mandarin speakers	Pearson's r=0.08, $t_{(33)}$=0.48, two-tailed, P=0.64, 95% CI [-0.26, 0.40]	Pearson's r=0.17, $t_{(33)}$=0.97, two-tailed, P=0.34, 95% CI [-0.18, 0.47]
	Future self-continuity vs. PD	Past self-continuity vs. PD
English speakers	Pearson's r=-0.25, $t_{(38)}$=-1.62, two-tailed, P=0.11, 95% CI [-0.52, 0.06]	Pearson's r=-0.09, $t_{(38)}$=-0.56, two-tailed, P=0.58, 95% CI [-0.39, 0.23]
Mandarin speakers	Pearson's r=-0.06, $t_{(38)}$=-0.40, two-tailed, P=0.69, 95% CI [-0.37, 0.25]	Pearson's r=0.12, $t_{(38)}$=0.76, two-tailed, P=0.45, 95% CI [-0.20, 0.42]
	Future self-continuity vs. Log k	Past self-continuity vs. Log k
English speakers	Pearson's r=-0.003, $t_{(37)}$=-0.02, two-tailed, P=0.99, 95% CI [-0.32, 0.31]	Pearson's r=0.12, $t_{(37)}$=0.76, two-tailed, P=0.45, 95% CI [-0.20, 0.42]
Mandarin speakers	Pearson's r=-0.19, $t_{(33)}$=-1.13, two-tailed, P=0.27, 95% CI [-0.49, 0.15]	Pearson's r=-0.06, $t_{(33)}$=-0.34, two-tailed, P=0.74, 95% CI [-0.38, 0.28]

Supplementary Table 7: Correlations between self-report and task data. We did not observe any associations between state or trait stress and psychological distance or discount rate in either English or Mandarin speakers. Likewise, we did not find any associations between future or past self-continuity and psychological distance or discount rate in either culture. Correlations between the measures and psychological distance included the full sample (n=40 English, n=40 Mandarin), while correlations between the measures and discount rate included the sample after discounting exclusions (n=39 English, n=35 Mandarin).

	(PD further-rated events – PD closer-rated events) vs. Log k
Across cultures	Pearson's r=-0.05, $t_{(72)}$=-0.39, two-tailed, P=0.70, 95% CI [-0.27, 0.18]
English speakers	Pearson's r=-0.05, $t_{(37)}$=-0.33, two-tailed, P=0.75, 95% CI [-0.36, 0.27]
Mandarin speakers	Pearson's r=-0.09, $t_{(33)}$=-0.49, two-tailed, P=0.63, 95% CI [-0.41, 0.25]

Supplementary Table 8: Exploration of the association between PD differentials and discount rates. We explored whether individuals who more strongly differentiated, i.e. drew stronger mental boundaries between events that they rated as occurring in the "further" past and future and the "closer" past and future discounted future rewards more strongly. We did not find evidence for a correlation between the difference in PD between closer and further events and discount rate across cultures or in either culture individually. However, as shown in Supplementary Figure 2a, our participants interpreted more events as occurring in the closer past and future than as occurring in the further past and future. A follow-up study with a more balanced distribution of closer and further event interpretations may be able to better investigate this question.
